# Establishment of a Necroptosis-Related Prognostic Signature to Reveal Immune Infiltration and Predict Drug Sensitivity in Hepatocellular Carcinoma

**DOI:** 10.3389/fgene.2022.900713

**Published:** 2022-07-25

**Authors:** Huili Ren, Jianglin Zheng, Qi Cheng, Xiaoyan Yang, Qin Fu

**Affiliations:** ^1^ Department of Pharmacology, School of Basic Medicine, Tongji Medical College, Huazhong University of Science and Technology, Wuhan, China; ^2^ Department of Neurosurgery, Union Hospital, Tongji Medical College, Huazhong University of Science and Technology, Wuhan, China; ^3^ Hepatic Surgery Center, Tongji Hospital, Tongji Medical College, Huazhong University of Science and Technology, Wuhan, China; ^4^ Key Laboratory for Drug Target Research and Pharmacodynamic Evaluation of Hubei Province, Wuhan, China

**Keywords:** hepatocellular carcinoma, necroptosis, prognostic, immune microenvironment, chemosensitivity

## Abstract

**Background:** Hepatocellular carcinoma (HCC) is a common type of primary liver cancer and has a poor prognosis. In recent times, necroptosis has been reported to be involved in the progression of multiple cancers. However, the role of necroptosis in HCC prognosis remains elusive.

**Methods:** The RNA-seq data and clinical information of HCC patients were downloaded from The Cancer Genome Atlas (TCGA) and International Cancer Genome Consortium (ICGC) databases. Differentially expressed genes (DEGs) and prognosis-related genes were explored, and the nonnegative matrix factorization (NMF) clustering algorithm was applied to divide HCC patients into different subtypes. Based on the prognosis-related DEGs, univariate Cox and LASSO Cox regression analyses were used to construct a necroptosis-related prognostic model. The relationship between the prognostic model and immune cell infiltration, tumor mutational burden (TMB), and drug response were explored.

**Results:** In this study, 13 prognosis-related DEGs were confirmed from 18 DEGs and 24 prognostic-related genes. Based on the prognosis-related DEGs, patients in the TCGA cohort were clustered into three subtypes by the NMF algorithm, and patients in C3 had better survival. A necroptosis-related prognostic model was established according to LASSO analysis, and HCC patients in TCGA and ICGC were divided into high- and low-risk groups. Kaplan–Meier (K–M) survival analysis revealed that patients in the high-risk group had a shorter survival time compared to those in the low-risk group. Using univariate and multivariate Cox analyses, the prognostic model was identified as an independent prognostic factor and had better survival predictive ability in HCC patients compared with other clinical biomarkers. Furthermore, the results revealed that the high-risk patients had higher stromal, immune, and ESTIMATE scores; higher TP53 mutation rate; higher TMB; and lower tumor purities compared to those in the low-risk group. In addition, there were significant differences in predicting the drug response between the high- and low-risk groups. The protein and mRNA levels of these prognostic genes were upregulated in HCC tissues compared to normal liver tissues.

**Conclusion:** We established a necroptosis-related prognostic signature that may provide guidance for individualized drug therapy in HCC patients; however, further experimentation is needed to validate our results.

## Introduction

Hepatocellular carcinoma (HCC) is the second mortality malignancy globally (Sung et al., 2021). Chemotherapy and molecular targeted therapies are the treatment modalities for patients with advanced HCC (Daher et al., 2018; Medavaram and Zhang, 2018), but the 5-year survival rate of patients is still low. The poor prognosis of HCC is mainly related to tumor heterogeneity, metastasis, recurrence, drug resistance, and the lack of predictive biomarkers in response to the treatment (Llovet et al., 2012; Llovet et al., 2015; Faivre et al., 2020). Thus, it is necessary to exploit novel therapeutic targets and reliable prognostic models for medical decision making.

Necroptosis, a form of programmed inflammatory cell death, was originally discovered as a form of cell death independent of caspase (Degterev et al., 2005). The canonical necroptotic pathway was triggered by tumor necrosis factor receptor (TNFR) family proteins, toll-like receptor 3 (TLR3)/TLR4, and lipopolysaccharide (LPS) (Yan et al., 2022). Thereafter, receptor-interacting serine/threonine-protein kinase 3 (RIPK3) is recruited and phosphorylated by RIPK1 in the absence of caspase-8, which is then phosphorylated by MLKL. Phosphorylation of MLKL leads to its oligomerization, and then translocate to cell membrane and form large pores that lead to necroptotic cell death by allowing ion influx, membrane lysis, followed by the uncontrollable release of intracellular material (Sun et al., 2012; Zhao et al., 2012). In recent times, necroptosis has been found to be involved in tumorigenesis, tumor progression, metastasis, and tumoral immune response (Hitomi et al., 2008; Yatim et al., 2015; Jiao et al., 2018). However, the exact function of necroptosis in tumor remains debatable. Findings imply that the exact role of necroptosis is dependent on the type of cancer and the different stages of disease development. The key molecular RIPK3 of necroptosis is required for tumorigenesis, such as in breast tumors (Lin et al., 2020). Inhibition the signal pathway by silence RIPK3 or suppresses the activity of RIPK3 may abolish inflammatory responses which is critical in modulating tumor initiation and progression (Seifert et al., 2016; Jayakumar and Bothwell, 2019). While necroptosis may also have tumor-suppressive properties. For acute myeloid leukemia, downregulation of RIPK3 is associated with poor survival (Najafov et al., 2017). For HCC, necroptosis inhibition may enhance the accumulation polarization of M2 TAMs, contributing to tumorigenesis (Wu et al., 2020) and sorafenib resistance (Liao et al., 2021). The mRNA level of RIPK3 might be a biomarker in tumor progression (Han et al., 2020).

However, the role of necroptosis in HCC prognosis is still unclear. In this study, we focused on the genes related to necroptosis regulation and constructed a prognostic model based on necroptosis-related genes (NRGs). Furthermore, we explored the relationship between prognosis and immune profiles, tumor mutational burden (TMB), and applications in predicting drug sensitivity. Our findings provide some new ideas for the diagnosis and treatment of HCC.

## Materials and Methods

### Data Collection and Preprocessing

The mRNA sequencing (FPKM) data and corresponding clinical information of HCC patients were downloaded from The Cancer Genome Atlas (TCGA; /https://portal.gdc.cancer.gov/repository; including 370 HCC tissue samples and 50 adjacent normal tissue samples) and International Cancer Genome Consortium (ICGC; https://dcc.icgc.org/projects/LIRI-JP; including 231 HCC samples). The gene expression profiles were normalized using the Perl language (http://www.perl.org/). The clinicopathological characteristics of HCC patients in TCGA and ICGC cohorts were summarized in Table 1. A total of 69 NRGs were collected from prior studies (Supplementary Table S1).

**TABLE 1:** Characteristics of hepatocellular carcinoma (HCC) patients in the training and validation cohorts.

Characteristics		Training cohort TCGA (*n* = 370)	Validation cohort ICGC (*n* = 231)
Age	≤65	232	90
	>65	138	141
Gender	Female	121	61
	Male	249	170
WHO grade	G1–2	232	—
	G3–4	133	—
	Unknown	5	—
TNM stage	I–II	256	141
	III–IV	90	90
	Unknown	34	—

### Identification of Differentially Expressed and Prognosis-Related Necroptosis Genes

DEGs in tumor tissues and normal tissues were analyzed using the “limma” package in Bioconductor in the R software (version 4.1.0). DEGs were identified based on the following criteria: false discovery rate (FDR) < 0.05 and a log2 fold change >1. Univariate analysis was applied in estimating the prognosis-related genes from the 69 NRGs. To analyze the correlation among the DEGs, a protein–protein interaction (PPI) network was built using the STRING (http://string-db.org/cgi/) online tool and the interaction score >0.2 was chosen as the cutoff criterion.

### Molecular Subtype Identification

We extracted the expression profiles of prognostic DEGs from the TCGA and ICGC databases. Thereafter, the nonnegative matrix factorization (NMF) clustering algorithm was used to cluster the HCC samples, the standard “brunet” option was selected, and 50 iterations were carried out. The number of clusters *k* was set to 2–10. According to indexes, including cophenetic, dispersion, and silhouette, the optimal number of clusters was finally determined. Kaplan–Meier (KM) survival curves were analyzed to compare the difference in survival rates among different groups.

### Construction of the Necroptosis-Related Gene Prognostic Model

The TCGA cohort was selected as the training cohort to construct the prognostic model. Univariate Cox analysis was applied to identify the NRGs that were significantly associated with HCC prognosis. Then, LASSO Cox regression analysis based on the “glmnet” R package was used to construct the prognostic signature. In time, the seven genes and their coefficients were retained, and the minimum criteria determined the penalty parameter (*λ*). The risk score of individual patients was also counted. Based on the median risk score value, the HCC patients in the TCGA and ICGC cohorts were divided into high- and low-risk groups, and ROC curves were utilized to predict the accuracy of prognostic signatures. Moreover, principal component analysis (PCA) and the T-distributed stochastic neighbor embedding (t-SNE)-based approach were adopted to validate the subtype assignments.

### Univariate and Multivariate Cox Regression Analysis

Univariate analysis was applied for estimating the associations between prognosis and age, gender, grade, stage, and risk score. Thereafter, multivariate analysis was presented for observing whether these factors were independently predictive of the prognosis of hepatocellular carcinoma. Hazard ratio (HR), 95% confidence interval (CI), and *p*-values were separately determined.

### Gene Ontology and the Kyoto Encyclopedia of Genes and Genomes Analyses

Gene Ontology (GO) and Kyoto Encyclopedia of Genes and Genomes (KEGG) pathway functional enrichment analyses were conducted using cluster Profiler R package (version 3.14.3) to assign various biological processes (BPs), molecular functions (MFs), cellular components (CCs), and pathways of identified marker genes in the interested cluster, and *p* < 0.05 was regarded as statistically enriched.

### Immune Cell Infiltration and Tumor Mutation Burden Analysis

The lollipop of immune responses is based on XCELL, TIMER, QUANTISEQ, MCPCOUNTER, EPIC, CIBERSORT-ABS, and CIBERSORT algorithms to analyze the Spearman correlation between risk score values and tumor-infiltrating immune cells. The tumor mutational data were downloaded from TCGA, and the “maftools” package was used to analyze the mutational data in both the high- and low-risk groups. The correlation between TMB and risk score was analyzed using the Pearson correlation test.

### Drug Response Prediction

The responses to regorafenib, cisplatin, tipifarnib, atezolizumab, gefitinib, sorafenib, erlotinib, axitinib, and bevacizumab were predicted by the Genomics of Drug Sensitivity in Cancer (https://www.cancerrxgene.org/) to analyze the relationship between the signature and drug response. We used pRRophetic R package (version 0.5) to compare the half-maximal inhibitory concentration (IC_50_) values between different risk groups by building a ridge regression model with 10-fold cross-validation.

### Human Hepatocellular Carcinoma Samples

Ten HCC tissue specimens and adjacent nontumorous tissues were obtained by surgery at Tongji Hospital of Tongji Medical College, and informed consent was obtained from the patients. All study methodologies were strictly in accordance with the Helsinki declaration for the use of human subjects and were approved by the Ethics Committee of Tongji Medical College, Huazhong University of Science and Technology.

### Realtime Quantitative PCR

Total RNA was extracted from HCC tissue using TRIzol Reagent (Life Technologies), and cDNA was generated using a PrimeScript RT reagent kit (TaKara, Japan). The RT-PCR reactions were followed according the instructions of SYBR^®^ Green Realtime PCR Master Mix (TaKara, Japan). The relative mRNA levels of target genes and housekeeping genes were calculated using the 2^−ΔΔCt^ method. All primers used in this study are listed in Supplementary Table S2.

### Statistical Analysis

Statistical analysis was conducted using R (version 4.1.0). Differential gene expression between two groups was identified using Wilcoxon rank sum test, with *p* value calculated for each gene. Pearson correlation analyses were performed to establish correlation coefficients. K–M survival analysis with log-rank test was used to assess survival differences between different groups. Data were depicted using the “ggplot2” package. The cutoff between high risk and low risk was determined using the “surv cutpoint” function in the “survminer” package, and all survival curves were visualized using the “survminer” package. *p* < 0.05 was considered statistically significant (*p < 0.05, **p < 0.01, ***p < 0.001).

## Results

### Identification of Prognosis-Related Necroptosis Genes in Hepatocellular Carcinoma

**FIGURE 1:**
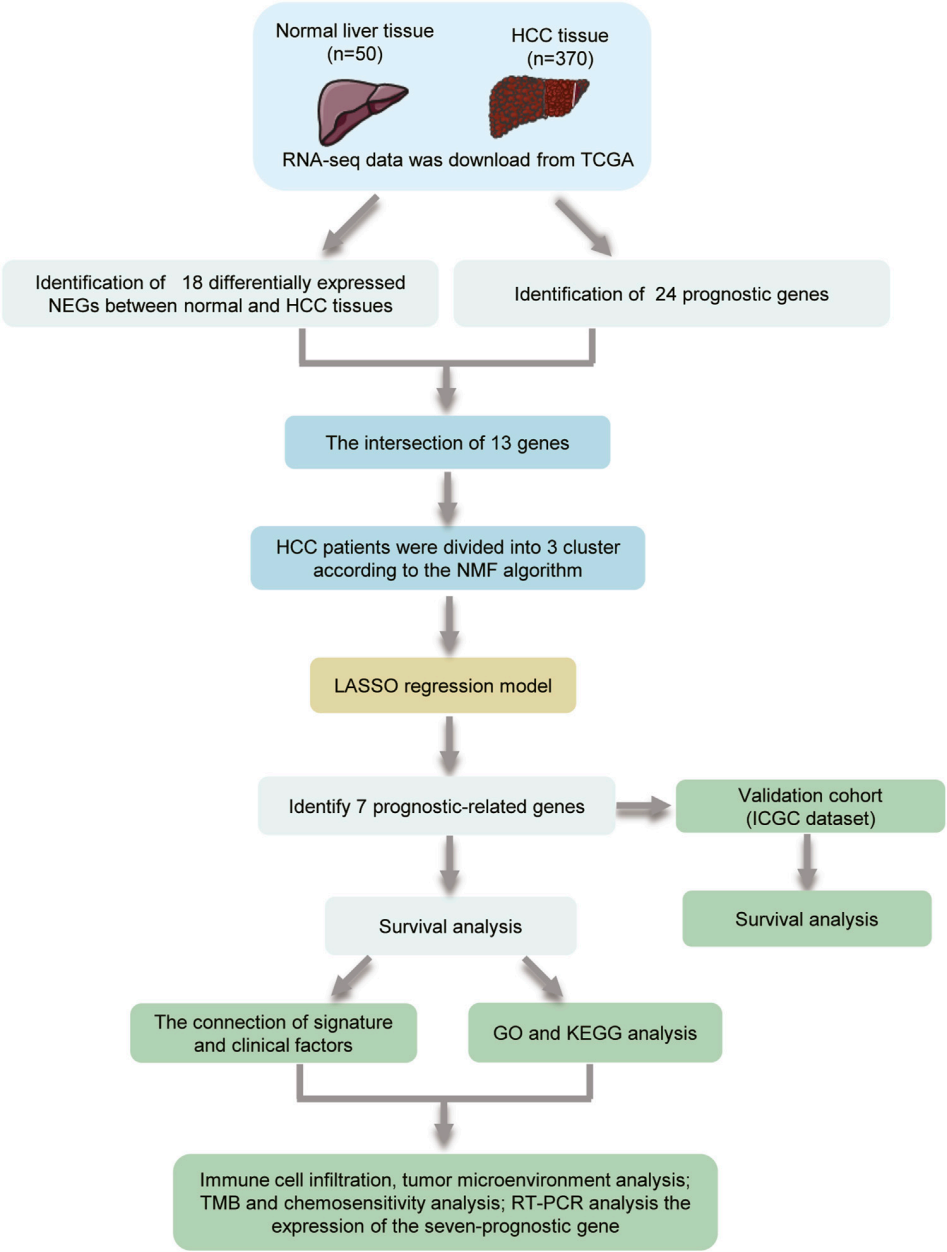
Flowchart showing the scheme of the study.

**FIGURE 2:**
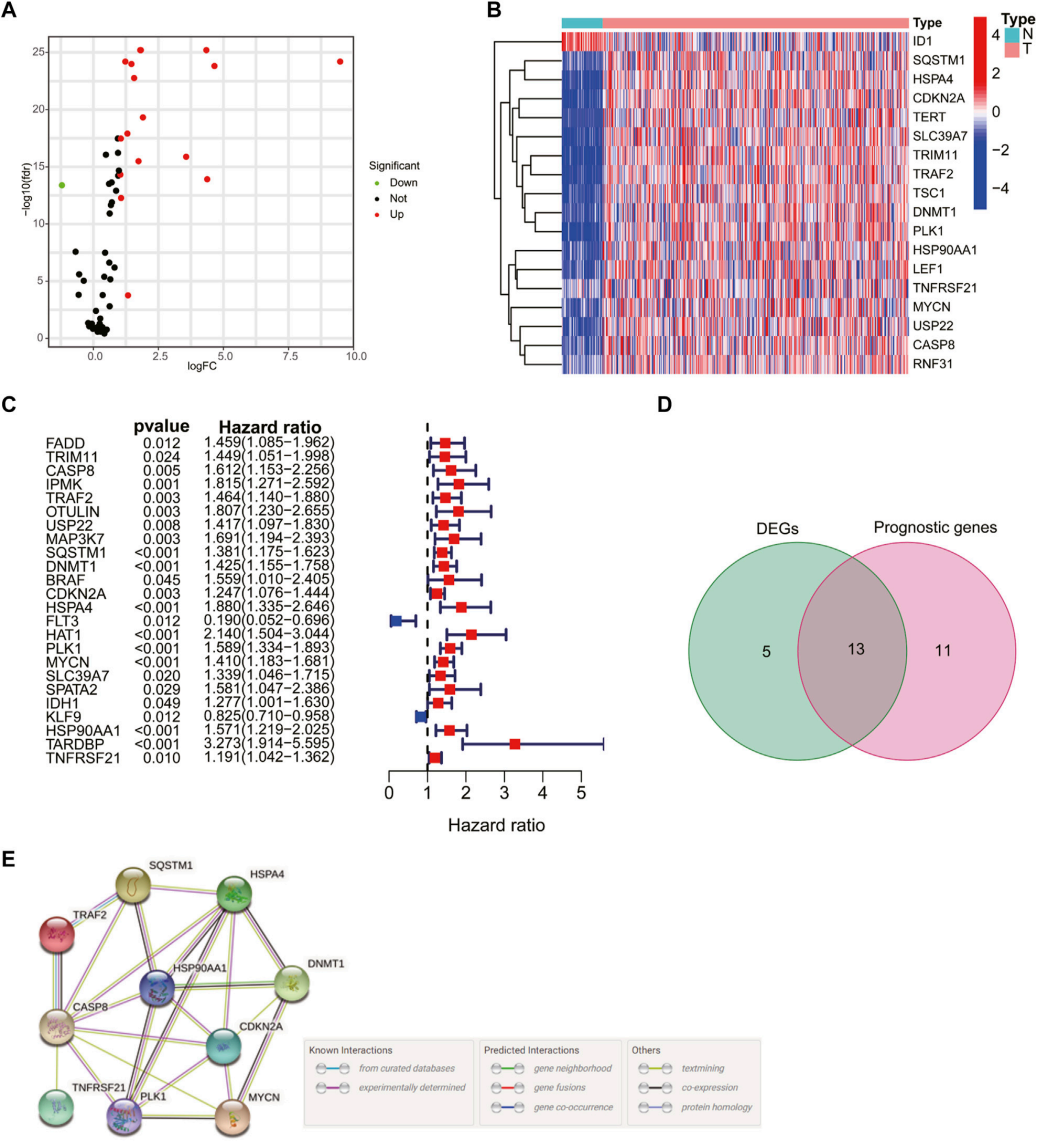
Identification of prognosis-related genes in hepatocellular carcinoma (HCC) patients. Volcano plot **(A)** and heatmap **(B)** of 18 differentially expressed necroptosis-related genes (NRGs). **(C)** Univariate Cox regression analysis of prognosis-related genes. **(D)** Venn plot showing the 13 intersection genes. **(E)** Protein–protein interaction network of the interactions among intersection genes.

The flowchart of the study is shown in Figure 1. A total of 69 NRG expression levels were evaluated in the TCGA cohort. Among the 69 NRGs, 18 DEGs were identified between normal and HCC samples as shown in the volcano plots and heatmap (Figures 2A, B; Supplementary Table S3), including 17 upregulated genes (*TSC1*, *TRIM11*, *CASP8*, *TRAF2*, *USP22*, *SQSTM1*, *DNMT1*, *CDKN2A*, *HSPA4*, *PLK1*, *MYCN*, *TERT*, *SLC39A7*, *RNF31*, *HSP90AA1*, *LEF1*, and *TNFRSF21*) and 1 downregulated gene (*ID1*). In addition, we determined 24 prognostic-related genes (PRGs) from the 69 NRGs by univariate Cox regression analyses (Figure 2C). Then, 13 prognostic DEGs were identified from the 18 DEGs and 24 PRGs (Figure 2D), and the PPI network provided interactive information among these 13 genes (Figure 2E).

### Molecular Typing Based on Differentially Expressed Genes

**FIGURE 3:**
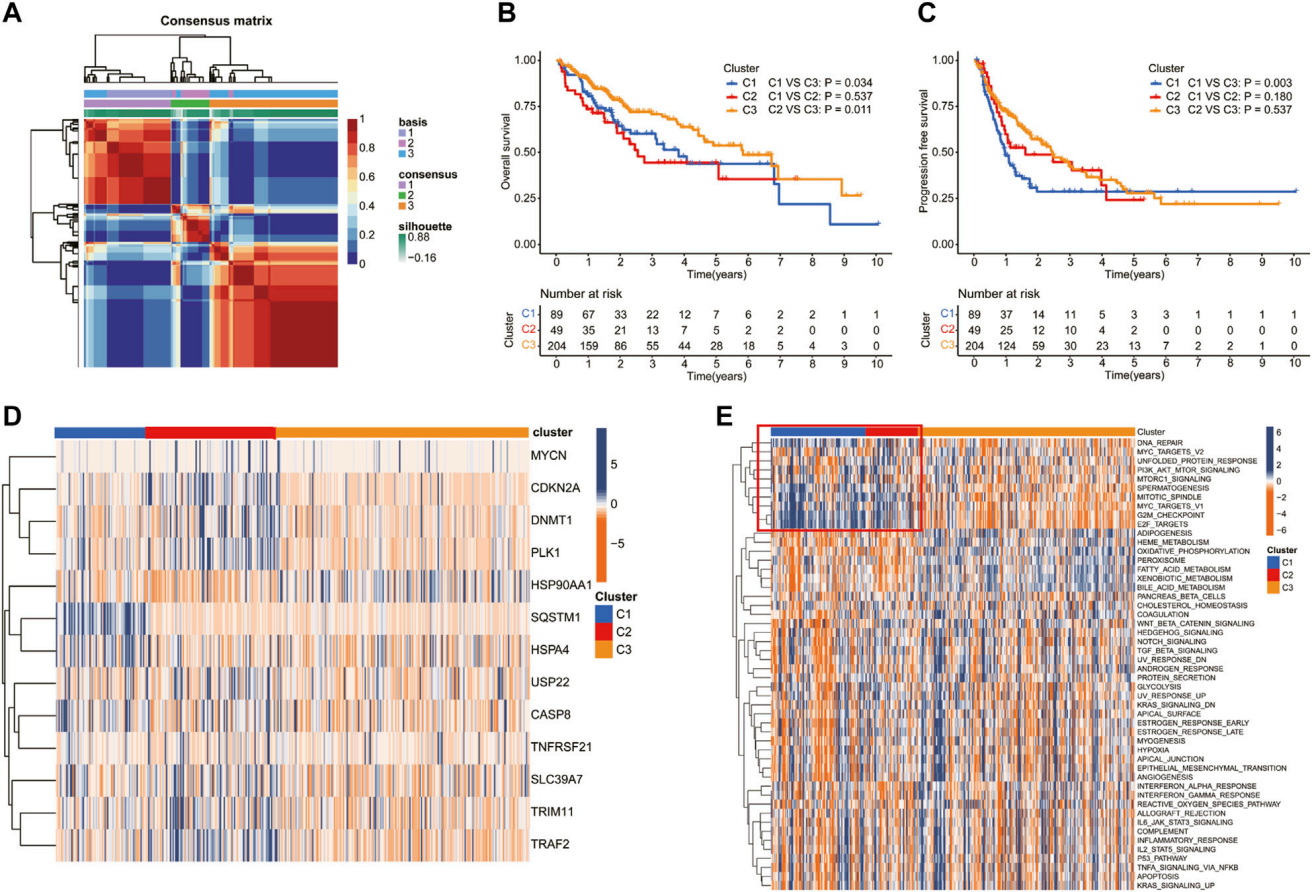
Screening of molecular subgroups through the nonnegative matrix factorization (NMF) cluster. **(A)** Consensus map of NMF clustering. The Kaplan–Meier (K–M) analysis of **(B)** overall survival and **(C)** progression-free survival for patients in different clusters. **(D)** Heatmap of the NRG expression of the molecular subtypes. **(E)** Heatmap showed the ssGSEA Z-scores of 50 hallmarks among the three clusters.

Patients in the TCGA cohort were divided into three clusters (C1, C2, and C3) according to the NMF algorithm (Figure 3A, Supplementary Figure S1). We compared the overall survival (OS) and progression-free survival (PFS) of the three clusters and found that C3 had better OS and PFS (Figures 3B, C). In addition, the expression of 13 prognostic DEGs among the three clusters was observed in the heatmap (Figure 3D). To explore the underlying molecular mechanism related to the three subtypes of HCC, we performed ssGSEA based on the transcriptome data of 50 gene sets retrieved from MSigDB. As shown in the Figure 3E, compared with C3, C1, and C2 were more correlated with the hallmark related to DNA repair, which may suggest an active proliferation of cancer cells.

### Establishment and Validation of an Necroptosis-Related Prognostic Model

**FIGURE 4:**
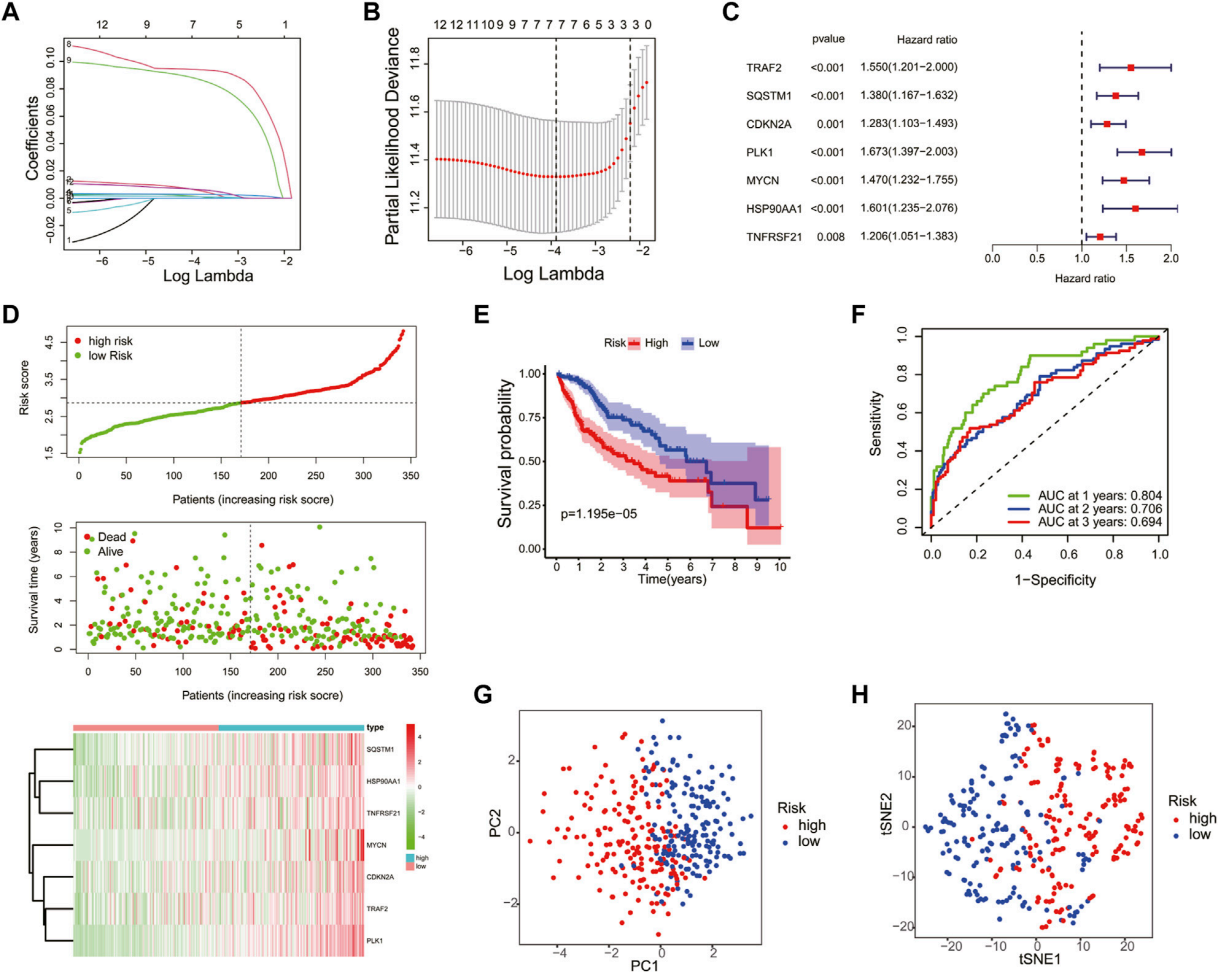
Construction of the necroptosis gene-based prognostic model in the Cancer Genome Atlas (TCGA) training cohort. The association between log(lamba) and coefficients of genes **(A)** and deviance **(B)**. **(C)** Univariate Cox regression analysis was used to construct a prognostic model. **(D)** Distribution of the risk scores, survival status, and expression of the four necroptosis-related risk genes in the training cohort. **(E)** The K–M curve of the low- and high-risk groups based on the seven-necroptosis-related gene profile. **(F)** Time-dependent ROC analysis for the 1-, 2-, and 3-year OS of the prognosis. PCA **(G)** and t-SNE **(H)** analysis of the TCGA cohort based on the risk score.

To construct a necroptosis-related prognostic signature, we performed LASSO regression analysis based on the prognostic significance of 13 DEGs in the TCGA cohort. According to the minimum criteria, a risk model consisting of *TRAF2*, *SQSTM1*, *CDKN2A*, *PLK1*, *MYCN, HSP90AA1*, and *TNFRSF21* was built (Figures 4A–C). The risk score was calculated using the following: risk score = (*TRAF2* × 0.0344 + *SQSTM1* × 0.2163 + *CDKN2A* × 0.0309 + *PLK1* × 0.3262 + *MYCN* × 0.2680 + *HSP90AA1* × 0.0917 + *TNFRSF21* × 0.0352). According to the median risk score, patients in the TCGA cohort were separated into high-risk (*n* = 170) and low-risk groups (*n* = 170). Moreover, patients in the high-risk group had a higher probability of death, increased expression level of the seven risk genes (Figure 4D), and a shorter OS (Figure 4E) compared to those in the low-risk group. In addition, the time-dependent ROC curve analysis demonstrated that this seven-gene prognostic model could predict the survival of the HCC patients (1-year AUC = 0.804, cutoff value: 2.560; 2-year AUC = 0.706, cutoff value: 2.959; and 3-year AUC = 0.694, cutoff value: 2.590) (Figure 4F). The PCA and t-SNE plot confirmed that the necroptosis-related prognostic model could separate high-risk patients from low-risk patients in the TCGA cohort (Figures 4G, H).

The efficiency of the risk model was validated in the ICGC cohort. Patients in the ICGC validation cohort were split into the high- and low-risk groups according to the median risk score (Supplementary Figure S2A). Similar to the TCGA cohort, patients in the high-risk group had higher death and expression of risk genes in the ICGC cohort (Supplementary Figure S2A). Moreover, the K–M curve showed that the low-risk group had a higher OS (Supplementary Figure S2B), and the ROC curve analysis confirmed the potent capability of the risk model to predict the survival of the patients in the ICGC cohort (1-year AUC = 0.684, cutoff value: 3.449; 2-year AUC = 0.718, cutoff value: 3.477; and 3-year AUC = 0.671, cutoff value: 3.718) (Supplementary Figure S2C). At last, the PCA and t-SNE plot indicated that the prognostic model could also separate the two risk groups in the ICGC cohort (Supplementary Figures S2D–E).

### Risk Score Is an Independent Prognostic Factor for Hepatocellular Carcinoma

**FIGURE 5:**
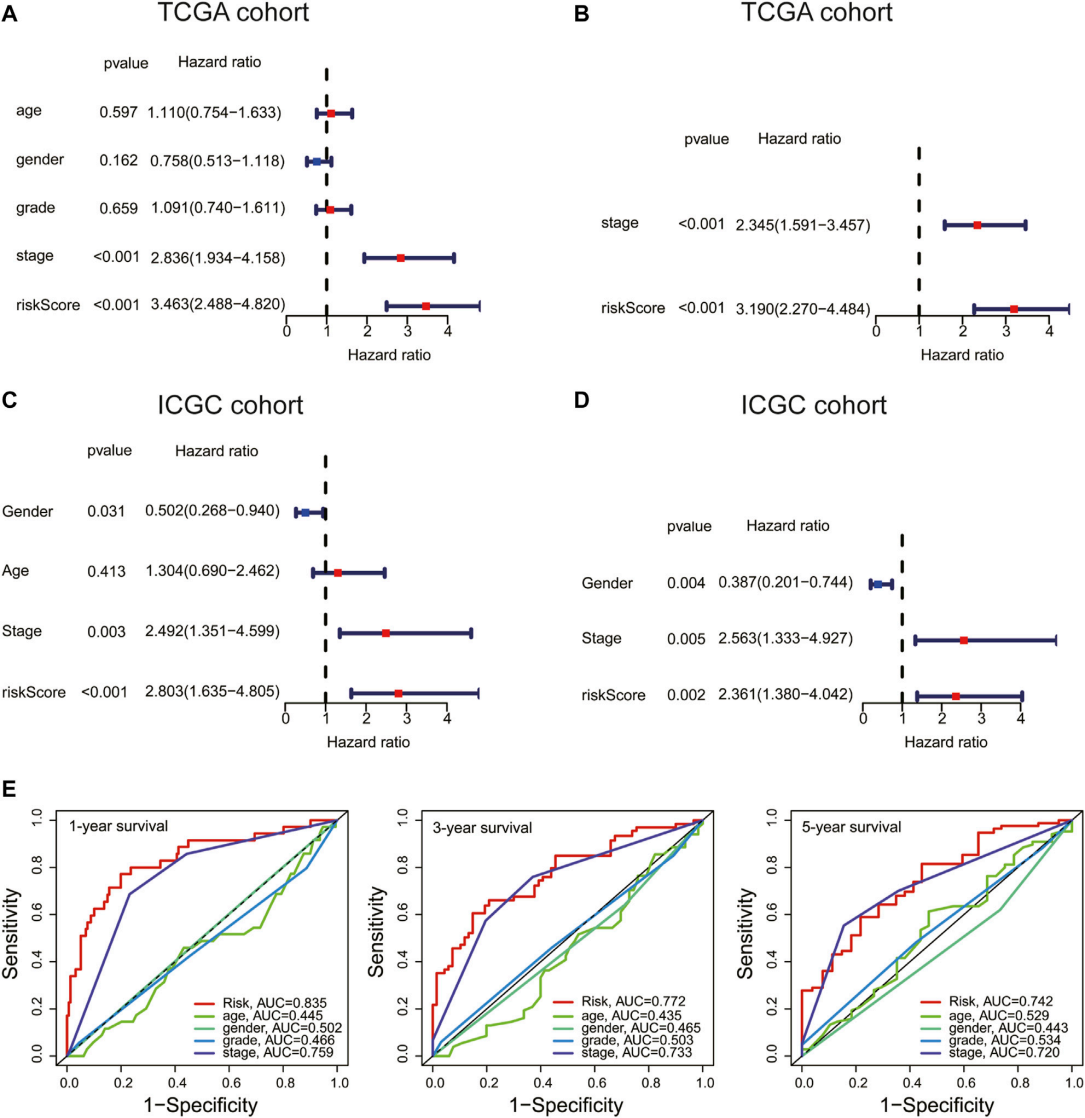
Risk score is an independent prognostic factor for HCC. **(A,C)** Univariate and **(B,D)** multivariate Cox analyses of the risk score and clinical variables in the TCGA and International Cancer Genome Consortium (ICGC) cohort. **(E)** ROC curve analysis of the risk score and clinicopathological characteristics in predicting 1-, 3-, and 5-year survival rates.

Univariate and multivariate Cox analyses were used to check whether the risk score could serve as an independent and robust biomarker to predict OS in HCC patients. As shown in Figure 5A, univariate Cox regression analysis showed that the risk score and stage were significantly correlated with the OS of HCC patients in the TCGA cohort. Thereafter, the risk score and stage were further identified as independent prognostic factors of OS by multivariate analysis (Figure 5B) (stage, HR = 2.345, 95% CI = 1.591–3.457, *p* < 0.001; risk score, HR = 3.190, 95% CI = 2.270–4.484, *p* < 0.001). In the ICGC cohort, the risk score was also confirmed as an independent prognostic factor (risk score, HR = 2.361, 95% CI = 1.380–4.042, *p* = 0.002) (Figures 5C, D). Furthermore, the accuracy of the risk score was highest in predicting 1-year, 3-year, and 5-year survival rates compared with other clinicopathological characteristics (Figure 5E). These results indicated that the risk score could be used as an independent prognostic factor to predict patient survival.

### Relationship Between the Risk Score and Clinical Factors

**FIGURE 6:**
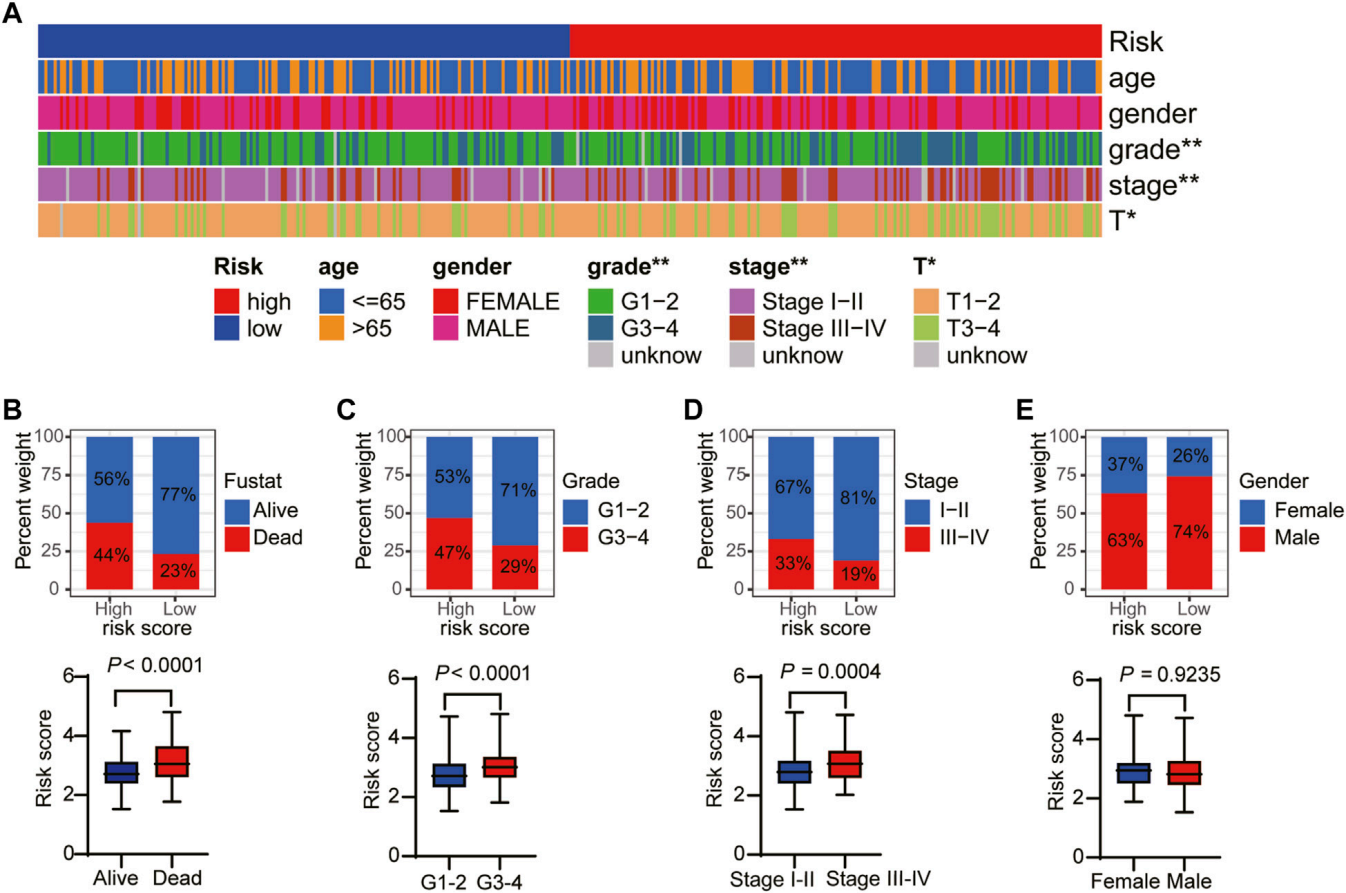
The relationship between the risk score and clinical factors. **(A)** The overview of the correspondence between necroptosis-related prognostic and other features of HCC patients. The proportion of patients with alive and death **(B)**, G1–G2 and G3–G4 **(C)**, stage I–II and III–IV **(D)**, and female and male **(E)**.

To explore the connection between the necroptosis-related prognostic model and clinical factors, we separated the patients in the TCGA dataset into several subgroups according to the different clinical parameters. The K–M curve showed that the high-risk patients had a poorer survival probability compared to the low-risk patients under the conditions of age > 65, age ≤ 65, G1–G2, G3–G4, stages I–II, III–IV, T1–T2, and T3–T4 (Supplementary Figures S3A–H). Furthermore, the heatmap exhibited the relationship between the high- and low-risk groups and the clinical factors, including age, gender, grade, and T stage (Figure 6A). Our further analysis found that in the high-risk group, a higher proportion of patients died and there was a higher proportion of late-stage patients (Figures 6B–D). While, the high-risk group had a lower proportion of male than the low-risk group (Figure 6E). 

### Gene Ontology Enrichment and Kyoto Encyclopedia of Genes and Genomes Pathway Analyses

**FIGURE 7:**
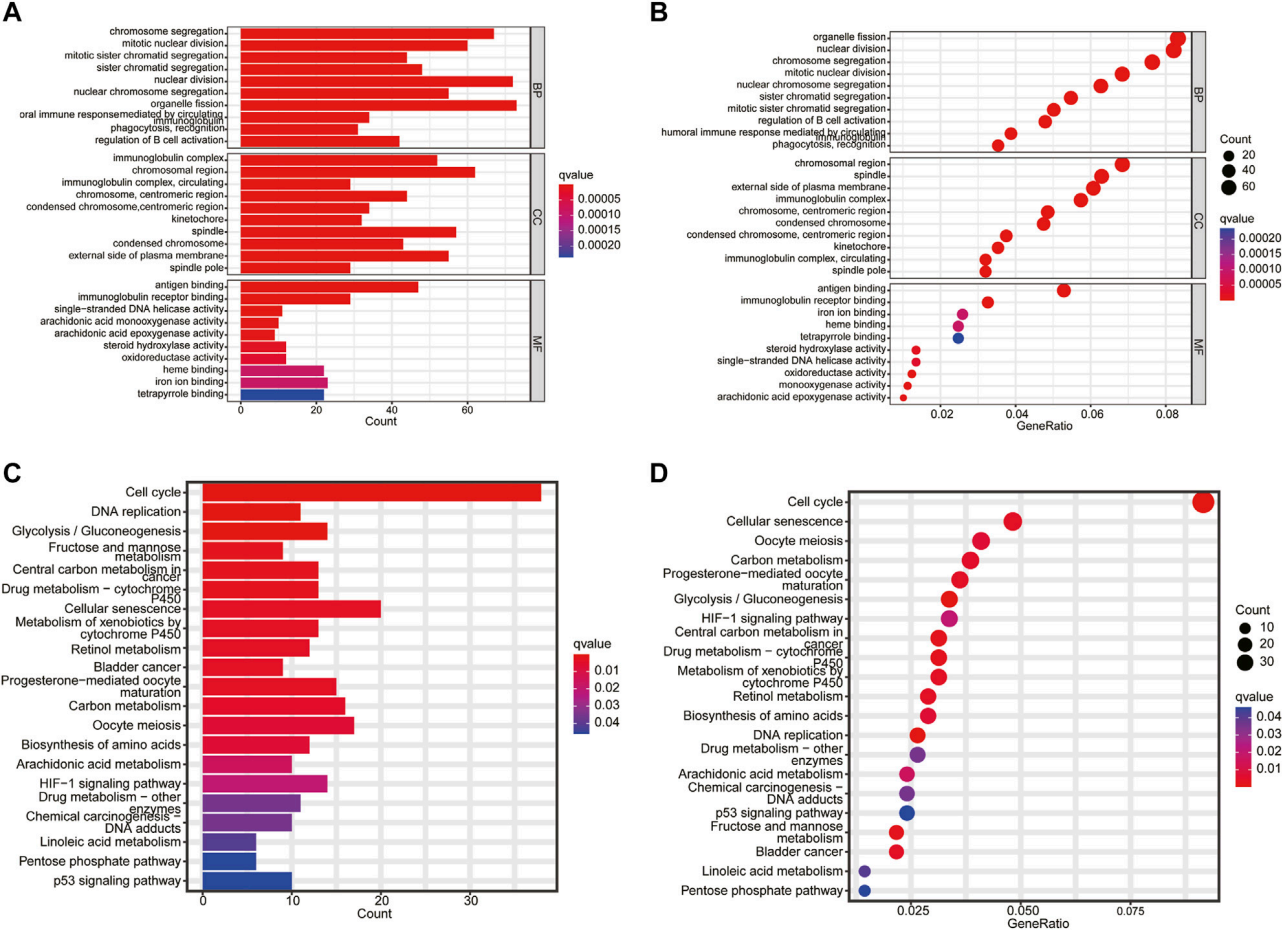
Functional enrichment analysis. **(A,B)** The top 10 biological process (BP) terms, CC terms, and MF terms of gene ontology analysis. **(C,D)** Kyoto Encyclopedia of Genes and Genomes enrichment analysis indicating related genes and pathways.

The biological functions and pathways related to the necroptosis-related prognostic model were measured by GO and KEGG analyses based on the DEGs between the high- and low-risk groups. GO analysis suggested that DEGs were mainly involved in chromosome segregation, mitotic nuclear division, humoral immune response, and B cell activation (Figures 7A, B). The KEGG results indicated that a number of DEGs were involved in the cell cycle, DNA replication, drug metabolism, and p53 signaling pathway (Figures 7C, D). These results demonstrated that the necroptosis-related prognostic signature may have a closed connection with the immune cell infiltration, tumor cell mutation, and metabolism of chemotherapeutic drugs.

### Correlation of Immune Cell Infiltration and Tumor Mutational Burden With Prognostic Signature

**FIGURE 8:**
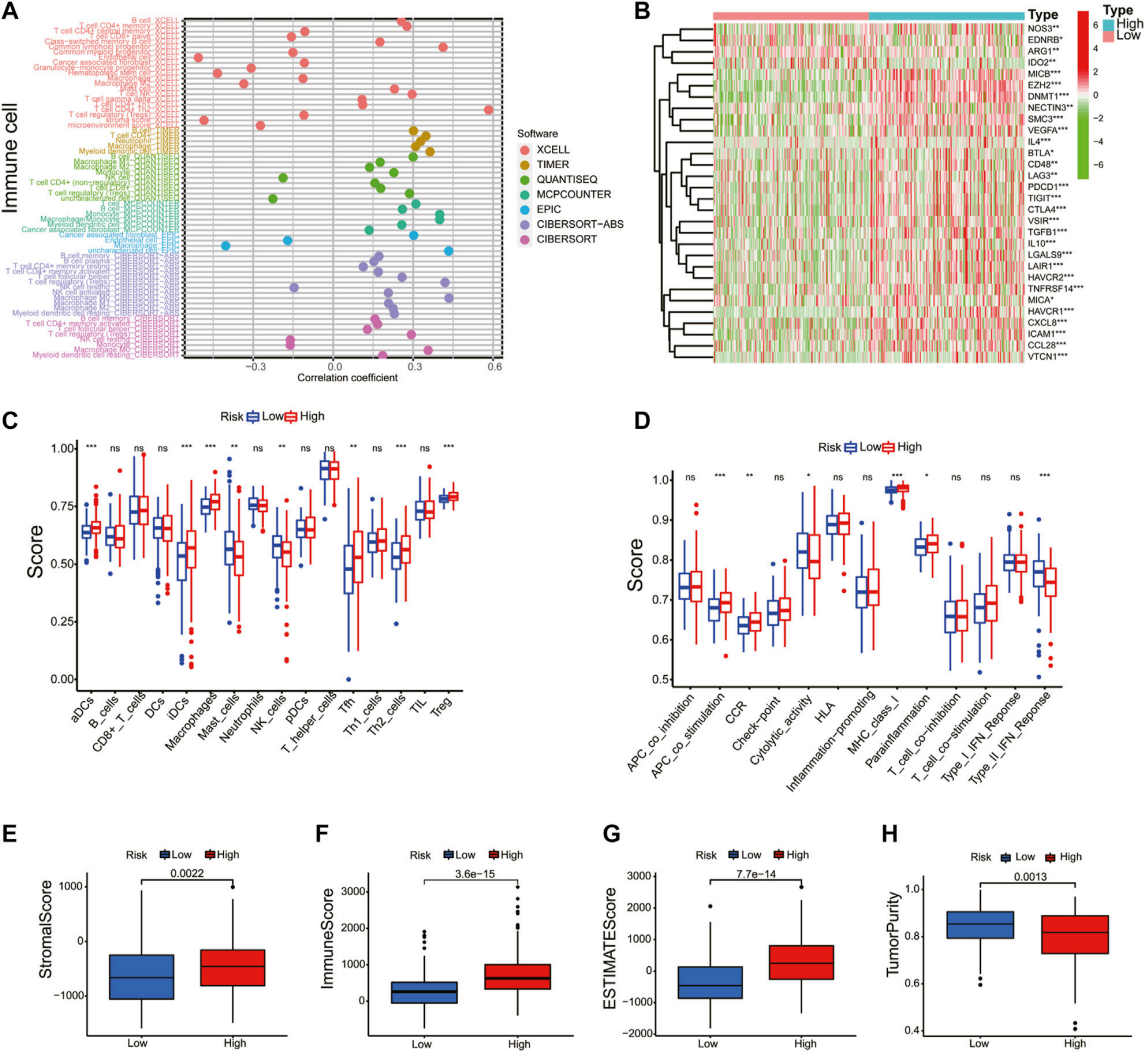
Correlation of immune cell infiltration and tumor mutational burden with prognostic signature. **(A)** A detailed Spearman correlation analysis was also performed using different algorithms. **(B)** The heatmap revealed the immune-related genes between the high- and low-risk groups. The activity differences of immune cells **(C)** and immune function **(D)** between the high- and low-risk groups. The immune score **(E)**, stromal score **(F)**, ESTIMATE score **(G)**, and tumor purity **(H)** were compared in the high- and low-risk groups.

Given the results of the GO and KEGG analyses, the XCELL, TIMER, QUANTISEQ, MCPCOUNTER, EPIC, CIBERSORT-ABS, and CIBERSORT algorithms were applied to explore the correlation between the prognostic model and immune cells. As shown in Figure 8A, the risk score was positively correlated to B cell and T-cell CD4^+^ memory and negatively correlated to the infiltration macrophage and T-cell regulatory (Tregs). Next, the immune-related genes that were significantly different in the high- and low-risk groups were exhibited in the heatmap (Figure 8B). Thereafter, ssGSEA analysis showed that antigen-presenting cell costimulation, CCR, MHC class I, and parainflammation were more highly activated in HCC patients with high-risk scores and cytolytic activity and type II IFN response were more active in the low-risk group (Figures 8C, D). At last, we analyzed the prognostic model on the HCC tumor immune microenvironment. Patients in the high-risk group had a higher immune score, stromal score, and ESTIMATE score, and lower tumor purity (Figures 8E–H). These findings revealed that the high-risk group might have more immune cells. Considering the crucial role of immune checkpoints in immunotherapies, the expression of immune checkpoints was analyzed between the high- and low-risk groups. As shown in Supplementary Figure S4, the expressions of *CD40*, *CD44*, *CD80*, *CD86*, *CD200*, *CD200R1*, *PD-L1*, *LAG3*, *HAVCR2*, *LAIR1*, *LGALS9*, *TNFSF18*, *TNFSF4*, *VTCN1*, *CTLA4*, and *NRP1* were remarkably higher in the high-risk group, suggesting that the high-risk patients might have a better response to immunotherapy.

TMB is a biomarker that indicates a response to immunotherapy. In this study, the relation between the risk score and TMB was investigated. As shown in Supplementary Figures S5A–B, the top 20 driver genes with the highest alteration of TP53 were significantly different between the high- and low-risk groups. Meanwhile, the K–M survival curves revealed that the high-TMB group demonstrated a poor prognosis (Supplementary Figure S5C). Moreover, we found that patients with high TMB and a high-risk score had a poorer prognosis (Supplementary Figure S5D). These results indicate that the prognostic model based on the seven NRGs could reflect the genomic stability of patients with HCC.

### Application of the Prognostic Model in Drug Sensitivity

**FIGURE 9:**
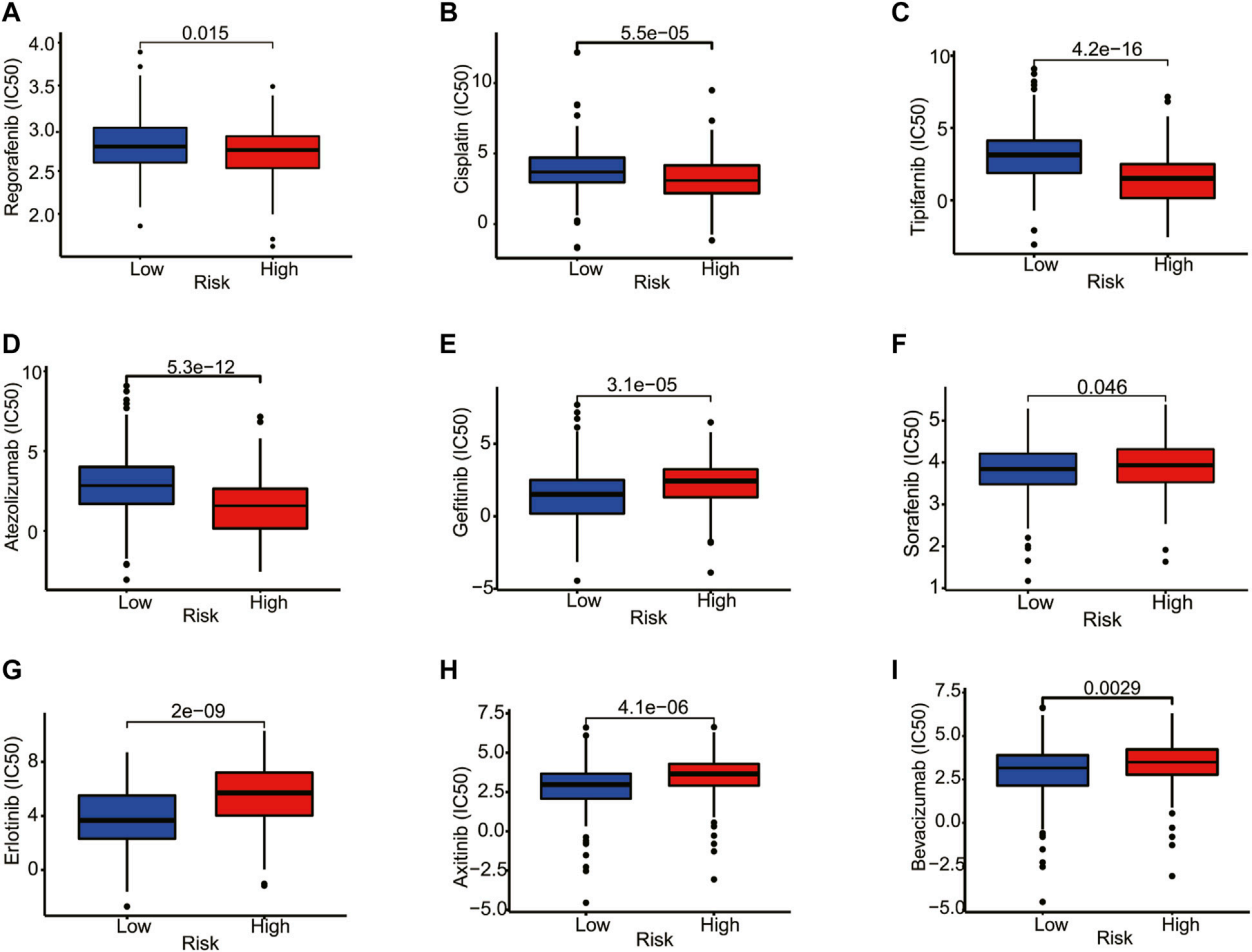
Correlation analysis between the estimated IC_50_ values of chemotherapy drugs and the risk score in HCC patients from the TCGA database. **(A)** Regorafenib, **(B)** Cisplatin, **(C)** Tipifarnib, **(D)** Atezolizumab, **(E)** Gefitinib, **(F)** Sorafenib, **(G)** Erlotinib, **(H)** Axitinib, **(I)** Bevacizumab.

Next, we explored the association between risk score and the efficacy of common chemotherapy drugs for HCC using IC_50_. The results revealed that several drugs in high-risk patients had lower IC_50_ values, including regorafenib, cisplatin, tipifarnib, and atezolizumab (Figures 9A–D, *p* < 0.05). However, gefitinib, sorafenib, erlotinib, axitinib, and bevacizumab were more sensitive to the patients in the low-risk group (Figures 9E–I, *p* < 0.05). Altogether, this result suggested the possibility of the prognostic model as a predictor of drug sensitivity.

### Expression of Prognostic Differentially Expressed Genes

**FIGURE 10:**
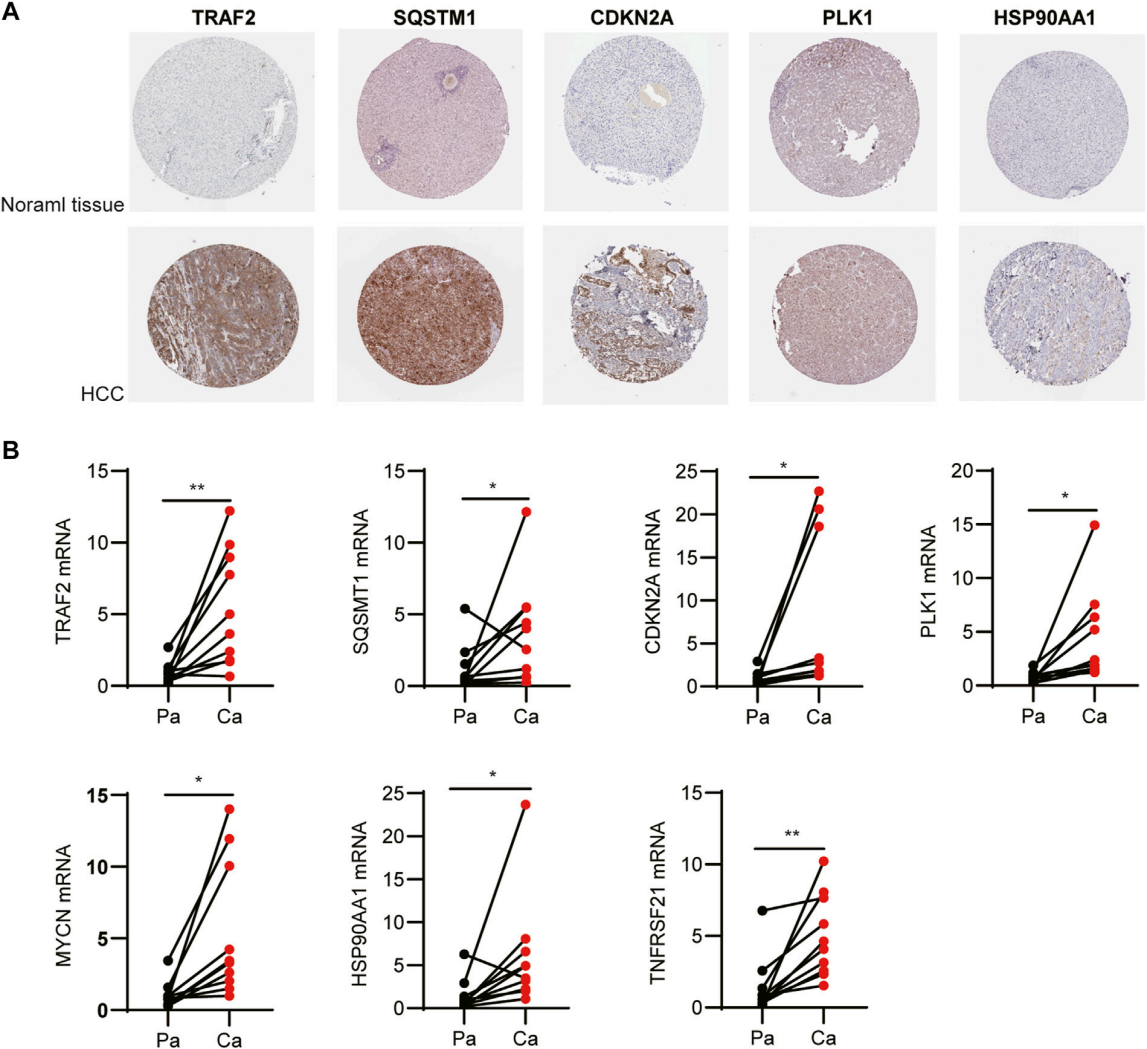
Expression of the independent prognostic genes. **(A)** Immunohistochemistry of *TRAF2*, *SQSTM1*, *CDKN2A*, *PLK1*, and *HSP90AA1* in the normal and tumor groups from the Human Protein Atlas database. **(B)** The mRNA levels of *TRAF2*, *SQSTM1*, *CDKN2A*, *PLK1*, *MYCN*, *HSP90AA1*, and *TNFRSF21* were measured by RT-PCR.

We examined the protein levels of these seven genes in risk models using the Human Protein Atlas (HPA) database. The results showed that the HCC tissues had higher protein levels of *TRAF2*, *SQSTM1*, *CDKN2A*, *PLK1*, and *HSP90AA1* (Figure 10A). While, *MYCN* and *TNFRSF21* were not found in HPA database. At last, to better validate the results of the bioinformatic analysis, the mRNA levels of *TRAF2*, *SQSTM1*, *CDKN2A*, *PLK1*, *MYCN*, *HSP90AA1*, and *TNFRSF21* obtained from the HCC patients were measured by RT-PCR. As expected, these seven genes were upregulated in the tumor tissues compared to the tumor-adjacent tissue (Figure 10B).

## Discussion

It has reported that necroptosis was associated with aggressive tumorigenesis and was thought to be an indication of poor prognosis (Richards et al., 2011; Caruso et al., 2012). However, owing to the lack of knowledge about the molecular mechanism of necroptosis in different types of cancer, the exact function of necroptosis in tumor development remains elusive (Yan et al., 2022). In the present study, we identified 13 prognosis-related NRGs in HCC. According to the NMF clustering, patients with HCC were divided into three clusters. C3 had better survival based on OS and PFS analyses compared with C1 and C2. To further evaluate the prognostic value of the DEGs, we constructed a necroptosis-related prognostic model using univariate Cox analysis and LASSO Cox regression analysis, and patients in the TCGA and ICGC cohorts were divided into high- and low-risk groups. We found that patients in the high-risk group had a worse prognosis compared to patients in the low-risk group in both the TCGA and ICGC cohorts. Moreover, the prognosis signature was proven to be an independent prognostic factor to predict the OS by univariate and multivariate Cox regression analyses. These results confirmed that our risk score may be stable as a predictor of HCC patient survival.

The necroptosis-related prognostic signature was composed of seven genes (*TRAF2*, *SQSTM1*, *CDKN2A*, *PLK1*, *MYCN*, *HSP90AA1*, and *TNFRSF21*), which had been confirmed to be closely related to tumorigenesis and necroptosis. *TRAF2*, as an adaptor molecule, was related with multiple receptor-specific functions in tumorigenesis and progression (Borghi et al., 2016). Moreover, *TRAF2* was identified as an important suppressor of necroptosis by directly binding to *MLKL* and recruiting cIAP1/2 (Karl et al., 2014; Petersen et al., 2015; Li et al., 2019). *SQSTM1*, also known as P62, is a multidomain scaffold protein with well-established roles in autophagy and tumor necrosis factor alpha (TNFα)- and NF-κB-related signaling pathways (Kirkin et al., 2009; Cha-Molstad et al., 2017; Cha-Molstad et al., 2018). However, researchers are increasingly finding that *SQSTM1* has an essential and complex role in tumor progression (Cuyler et al., 2022). Elevated *SQSTM1* expression had been reported to support tumorigenesis (Guo et al., 2011). Using 40 cases of tumor tissue chip, elevated *SQSTM1* expression was mainly observed in the cytoplasm of pancreatic carcinoma cells and differently expressed among the T stages (Mohamed et al., 2015; Zhang et al., 2020). MLKL deficiency prevents the accumulation of *SQSTM1* in the liver (Wu and Nagy, 2020). For age-related ischemia/reperfusion, *SQSTM1* forms a complex with RIP1–RIP3, which contributes to myocardial necroptosis (Li et al., 2020). *CDKN2A*, also known as the *P16* gene, had a crucial role in the regulation of the cell cycle (Luo et al., 2021). Although various studies have classified *CDKN2A* as a tumor suppressor, some studies have identified its complex role in tumors (Rangel et al., 2022). It was reported that the expression of the *CDKN2A* gene was significantly higher in 15 tumors, and the *CDKN2A* expression level was significantly correlated with the TMB, microsatellite instability, and infiltrating lymphocyte (Chen et al., 2021). In addition, high *CDKN2A* expression and low FGFR3 expression were statistically significantly associated with worse PFS (Breyer et al., 2018) and were associated with a higher grade (Quentin et al., 2004; Ploussard et al., 2011; Abat et al., 2014). Given the multiple roles of *CDKN2A*, further studies need to be done to validate the effects of *CDKN2* in tumor progression. *PLK1* is a serine/threonine-protein kinase involved in cell cycle regulation and mitotic progression (Xie et al., 2005). Overexpression of *PLK1* was observed in prostate cancer (Weichert et al., 2004), colorectal cancer (Takahashi et al., 2003), neuroblastomas (Ramani et al., 2015), and rectal cancer (Tut et al., 2015) and was associated with poor prognosis. *PLK1* knockout suppressed cancer cell survival, induced apoptosis, and increased the sensitivity to chemotherapy drugs (Reagan-Shaw and Ahmad, 2005; Weiss and Efferth, 2012). Moreover, *PLK* was identified as one of the necroptosis-related prognosis genes of invasive breast carcinoma (Hu et al., 2022) and clear cell renal cell carcinoma (Xin et al., 2022). In addition, *PLK1* mediated the phosphorylation of RIPK3 by directly associated with RIPK3 as cell enter mitosis (Gupta and Liu, 2021). *MYCN*, one of the MYC families of oncogenes, was related to cell proliferation, cell adhesion, DNA repair, and metabolism (Arvanitis and Felsher, 2006; Chen et al., 2018). Studies found that 25% of patients with neuroblastoma showed an *MYCN* amplification and predicted poor prognosis independently of other factors (Brodeur et al., 1984; Seeger et al., 1985). *HSP90AA1*, one of the HSP90 isoforms, showed a significant correlation with survival time in lung cancer patients by inhibiting the AKT1 and ERK pathways (Niu et al., 2021) and was upregulated in colorectal cancer (Szczuka et al., 2021). *HSP90* regulates the stability of MLKL and RIP3 and is required for TNF-stimulated necrosome assembly (Zhao et al., 2016). *TNFRSF21* is the member of the TNF receptor superfamily. Studies suggested that *TNFRSF21* might serve as a prognostic marker for esophageal squamous cell carcinoma (Qiu et al., 2021) and esophagus adenocarcinoma (Zhang et al., 2021). Furthermore, it should be mentioned that the impact and mechanism of these seven NRGs in HCC have not been reported yet, and future experiments are needed to provide more evidence.

GO and KEGG analyses revealed that the gene sets of the high-risk patients were enriched in humoral immune response, DNA replication, and drug metabolism, which indicated that the necroptosis-related prognosis may be associated with the tumor immune environment and the sensitivity to chemotherapy drugs. In addition, necroptosis exhibited an important role in the regulation of the immune response. In melanoma, a high level of potassium was released from the necrotic tumor cell, which inhibits CD4^+^ and CD8^+^ T-cell activities, resulting in the blockade of antitumor immunity (Vodnala et al., 2019). Moreover, upregulation of RIPK1 in tumor-associated macrophages (TAMs) contributes to immune tolerance and immunotherapeutic resistance in pancreatic ductal adenocarcinoma (Wang et al., 2020). Further analyses indicated that the composition of immune cells was different between high- and low-risk groups. Our results confirmed that higher immune, stromal, and ESTIMATE scores, and lower tumor purity were observed in patients with a high-risk score, which provided further evidence revealing the connection between the tumor microenvironment and necroptosis. In addition, our study confirmed that the expression of multiple immune checkpoints showed considerable difference between the high- and low-risk groups, which may offer underlying therapeutic targets for HCC.

Another biomarker that has recently garnered significant attention is TMB, which demonstrated reasonable prediction of immunotherapy responses (Lauss et al., 2017). In the present study, we found that the high-TMB group demonstrated a poorer prognosis in HCC patients. More importantly, HCC patients with high TMB combined with a high-risk score had a poorer prognosis. The *TP53* gene encodes the tumor protein p53, which is a tumor suppressor that prevents cell division and proliferation. We found that TP53 mutation is related to a poor prognosis of HCC, and it has a high mutation rate in the high-risk group. Drug sensitivity prediction revealed that regorafenib, cisplatin, tipifarnib, and atezolizumab showed lower IC_50_ values in patients with high-risk scores. The results confirmed that prognosis can be used to predict drug sensitivity.

Some limitations must be addressed in our research. First, owing to the heterogeneity of the HCC tissue, more samples should be included in the future to ensure the stability and accuracy of signature prediction. Second, molecular mechanism was not characterized. Further experiments are needed to explore the interaction between HCC and NRGs. Third, the data in our research were obtained from public databases and lacked more basic experimental verification. At last, the complex interaction between HCC and immune cells in necroptosis remains to be further explored.

In conclusion, our study provided a new marker for predicting the prognosis of patients with HCC and provided an important basis for the further study of the relationship among NRGs, the immune microenvironment, and chemotherapy treatment in HCC.

## Data Availability

The original contributions presented in the study are included in the article/Supplementary Material. Further inquiries can be directed to the corresponding author.
