# Supplementary material for: Establishment of a Necroptosis-Related Prognostic Signature to Reveal Immune Infiltration and Predict Drug Sensitivity in Hepatocellular Carcinoma
Source: Front Genet. 2022 Jul 25;13:900713. doi: 10.3389/fgene.2022.900713 (PMC9357940; doi:10.3389/fgene.2022.900713)
Supplement: Supplementary file 1 [file DataSheet1.docx]

Supplementary Material

## Supplementary Figures


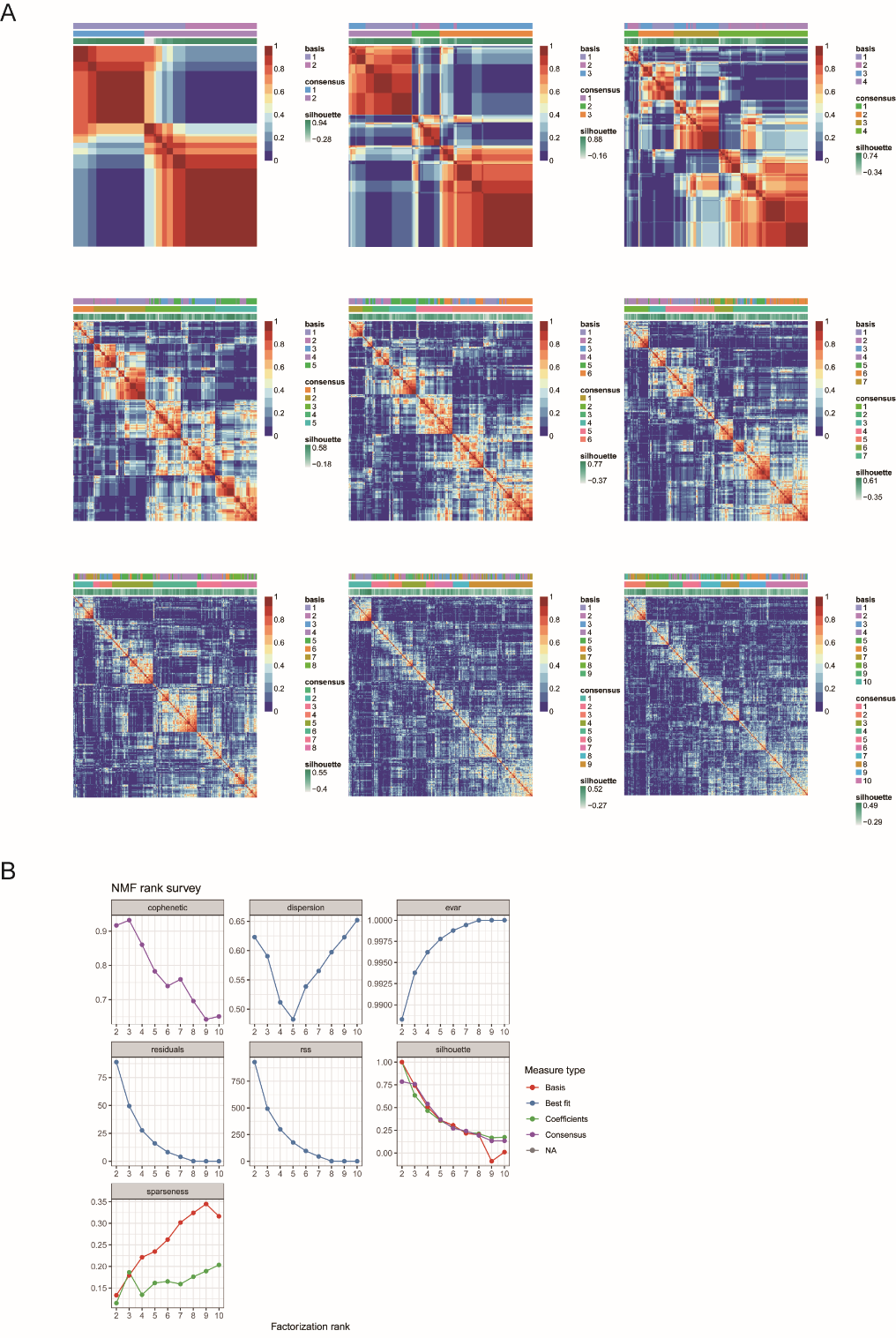


**Supplementary Figure 1.** The screening process of molecular subgroups through NMF cluster. **(A)** the heatmap of consensus matrix 2-10 subgroups. **(B)** the NMF rank survey results showing an optimal grouping of three subgroups.


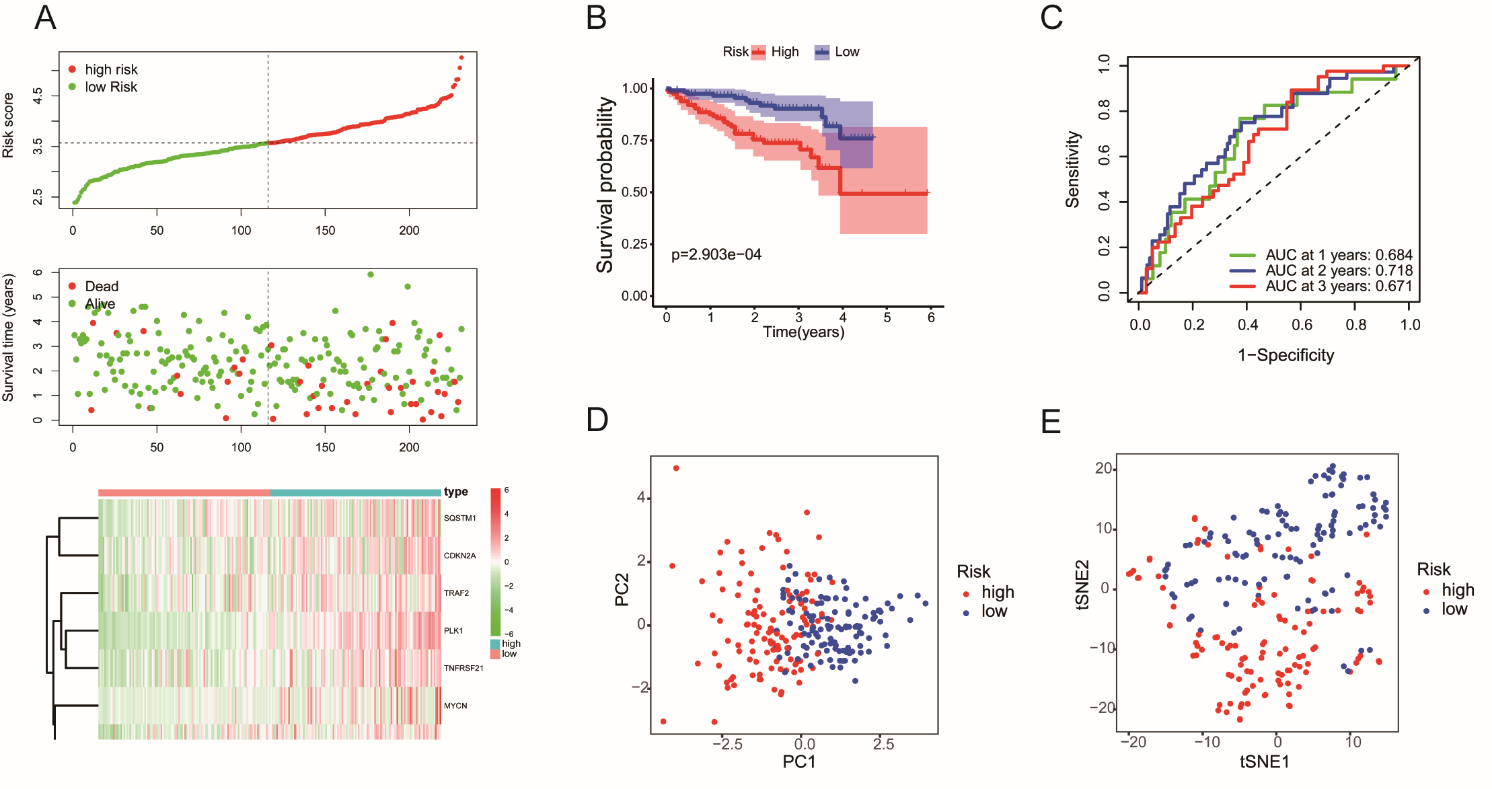


**Supplementary Figure 2.** External validation of the risk model. **(A)** Distribution of the risk scores, survival status, and expression of the seven necroptosis-related risk genes in the ICGC cohort. **(B)** The KM analysis of the overall survival in the high-risk and low-risk groups. **(C)** The ROC analysis to estimate the predictive efficiency. PCA and t-SNE analysis of the high-risk and low-risk groups in the ICGC cohort **(D-E)**.


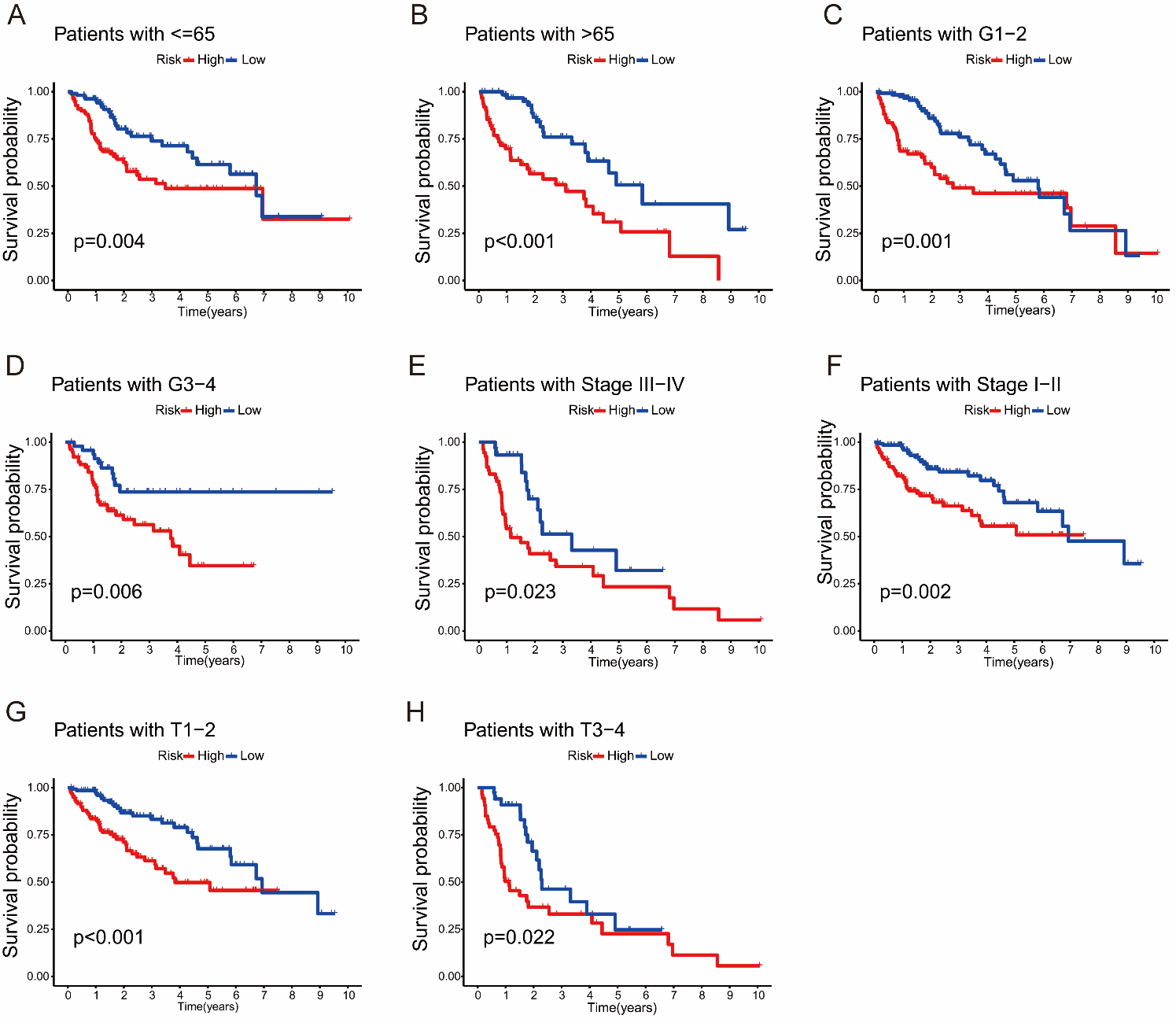


**Supplementary Figure 3.** The survival analysis based on the prognostic model.


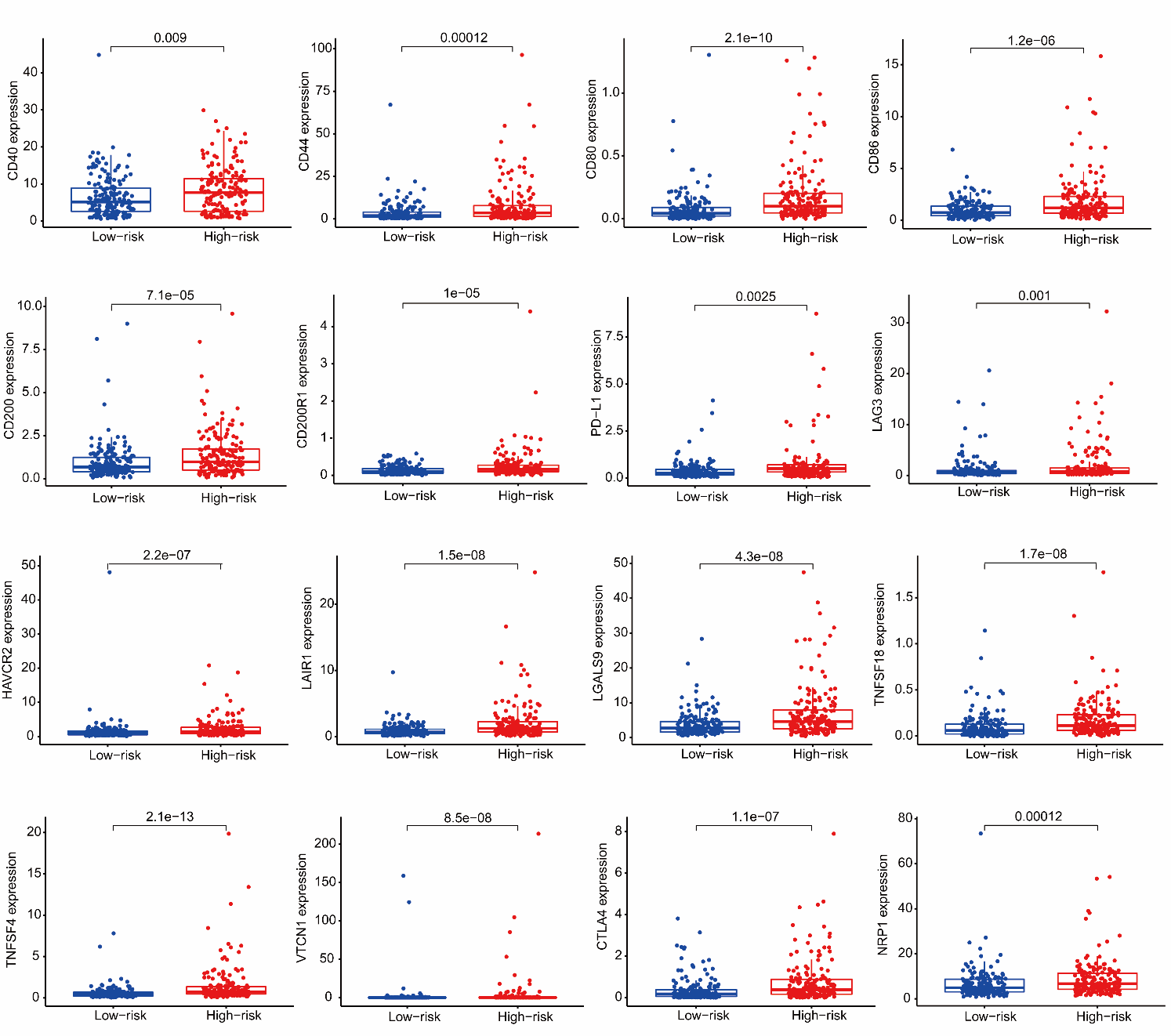


**Supplementary Figure 4.** The gene expression of immune checkpoints between two risk group.


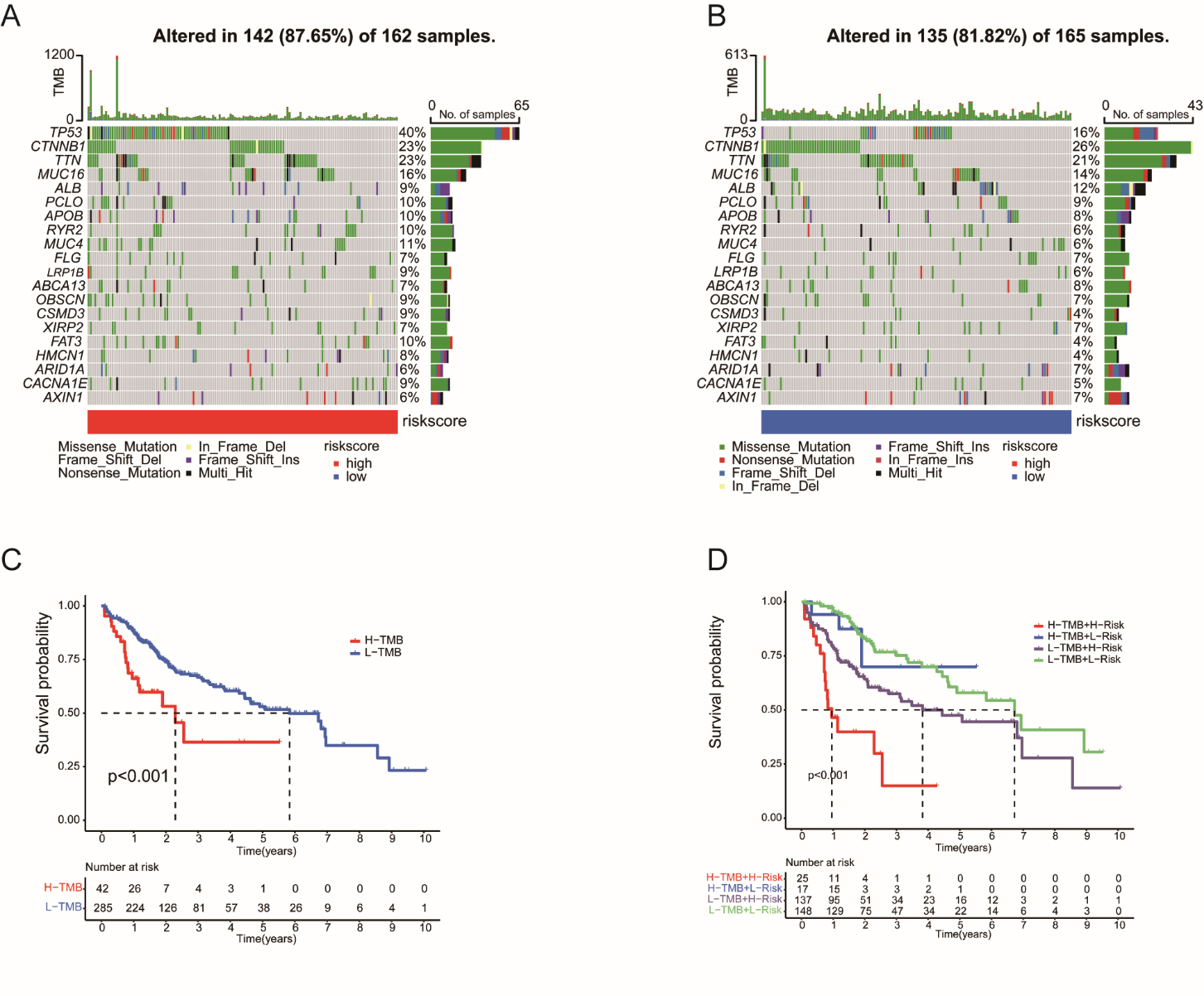


**Supplementary Figure 5.** The relationship between TMB and the risk score. The top 20 driver genes with the highest alteration in the high-risk group **(A)** and low-risk group **(B)**. **(C)** The KM curve of the low- and high-TBM groups. **(D)** Survival analysis of high/low TMB combined with high/low risk.

## Supplementary Tables

**Supplementary table 1.** The source of 69 necroptosis-related genes.

| Genes | Source |
| --- | --- |
| FADD | necroptosis geneset M24779.gmt |
| FAS | necroptosis geneset M24779.gmt |
| FASLG | necroptosis geneset M24779.gmt |
| MLKL | necroptosis geneset M24779.gmt |
| RIPK1 | necroptosis geneset M24779.gmt |
| RIPK3 | necroptosis geneset M24779.gmt |
| TLR3 | necroptosis geneset M24779.gmt |
| TNF | necroptosis geneset M24779.gmt |
| TSC1 | PMID: 31961824; PMCID: PMC7108921. |
| TRIM11 | PMID: 31961824; PMCID: PMC7108921. |
| CASP8 | PMID: 31748744. |
| ZBP1 | PMID: 31076724; PMCID: PMC7109092. |
| MAPK8 | PMID: 31706322; PMCID: PMC6842489. |
| IPMK | PMID: 29883610; PMCID: PMC5994928. |
| ITPK1 | PMID: 29883610; PMCID: PMC5994928. |
| SIRT3 | PMID: 31894331. |
| MYC | PMID: 32753382; PMCID: PMC7443878. |
| TNFRSF1A | PMID: 27049944; PMCID: PMC4833566. |
| TNFSF10 | PMID: 27049944; PMCID: PMC4833566. |
| TNFRSF1B | PMID: 26993379. |
| TRAF2 | PMID: 26993379. |
| PANX1 | PMID: 31410978; PMCID: PMC6776911. |
| OTULIN | PMID: 29950720. |
| CYLD | PMID: 28362430; PMCID: PMC5520167. |
| USP22 | PMID: 33369872; PMCID: PMC7857539. |
| MAP3K7 | PMID: 27219062; PMCID: PMC4886731. |
| SQSTM1 | PMID: 27219062; PMCID: PMC4886731. |
| STAT3 | PMID: 32100392; PMCID: PMC7171322. |
| DIABLO | PMID: 27194728. |
| DNMT1 | PMID: 32554751; PMCID: PMC7416440. |
| CFLAR | PMID: 30518925; PMCID: PMC6281604. |
| BRAF | PMID: 30157175; PMCID: PMC6114281. |
| AXL | PMID: 30157175; PMCID: PMC6114281. |
| ID1 | PMID: 32004572. |
| CDKN2A | PMID: 28811972; PMCID: PMC5543818. |
| HSPA4 | PMID: 32156734; PMCID: PMC7104336. |
| BCL2 | PMID: 33239070; PMCID: PMC7687715. |
| STUB1 | PMID: 29686306; PMCID: PMC5913227. |
| FLT3 | PMID: 30828789. |
| HAT1 | PMID: 29535128. |
| SIRT2 | PMID: 29535128. |
| SIRT1 | PMID: 29535128. |
| PLK1 | PMID: 22890325; PMCID: PMC3499666. |
| MPG | PMID: 30755477; PMCID: PMC7150588. |
| BACH2 | PMID: 31918262. |
| GATA3 | PMID: 31918262. |
| MYCN | PMID: 26633716; PMCID: PMC4720889. |
| ALK | PMID: 26633716; PMCID: PMC4720889. |
| ATRX | PMID: 26633716; PMCID: PMC4720889. |
| TERT | PMID: 26633716; PMCID: PMC4720889. |
| SLC39A7 | PMID: 30237509; PMCID: PMC6748104. |
| SPATA2 | PMID: 27545878; PMCID: PMC5009064. |
| RNF31 | PMID: 27545878; PMCID: PMC5009064. |
| IDH1 | PMID: 28564603. |
| IDH2 | PMID: 28564603. |
| KLF9 | PMID: 30348136; PMCID: PMC6198521. |
| HDAC9 | PMID: 30348136; PMCID: PMC6198521. |
| HSP90AA1 | PMID: 23147571. |
| LEF1 | PMID: 22157808. |
| BNIP3 | PMID: 20963496. |
| CD40 | PMID: 26313915; PMCID: PMC4558516. |
| BCL2L11 | PMID: 24561519. |
| EGFR | PMID: 25688715. |
| DDX58 | PMID: 33852834; PMCID: PMC8109599. |
| TARDBP | PMID: 33852834; PMCID: PMC8109599. |
| APP | PMID: 34105277; PMCID: PMC8188212. |
| TNFRSF21 | PMID: 34105277; PMCID: PMC8188212. |
| TRADD | PMID: 32039207 |

**Supplementary table 2.** The Primers sequence used in this study.

| MYCN | forward | GATGCACCCCCACAGAAGAA |
| --- | --- | --- |
|  | reverse | CTCCGAGTCAGAGTTTCGGG |
| QSTM1 | forward | GTCAATTTCCTGAAGAATGTGGG |
|  | reverse | GAGTTCACCTGTGG ATGGGTC |
| CDKN2A | forward | CCGCCGCGAGTGAGGGTTTT |
|  | reverse | CGCTGCCCATCATCATGACCTGG |
| PLK1 | forward | ATAGAGCGTGACGGCACTGAGT |
|  | reverse | TGCTCGCTCATGTAATTGCG |
| TRAF2 | forward | CTGCCCTTGCTGTCCTGT |
|  | reverse | CCCTCGTTTCTGCCTGTC |
| HSP90AA1 | forward | GTGTGCAACAGCTGAAGGAA |
|  | reverse | CTCTCCATGTTTGCTGTCCA |
| TNFRSF21 | forward | AAGCCGGGGACCAAGGAGACAGACAAC |
|  | reverse | TGCCGGGGCCCTTTTTCAGAGT |
| 18S | forward | TTGACGGAAGGGCACCACCAG |
|  | reverse | GCACCACCACCCACGGAATCG |

**Supplementary table 3.** Differential expression necroptosis-related genes.

| gene | conMean | treatMean | logFC | pValue | fdr |
| --- | --- | --- | --- | --- | --- |
| TSC1 | 0.723672 | 2.14458 | 1.567288 | 2.41E-24 | 1.72E-23 |
| TRIM11 | 0.753209 | 2.625952 | 1.801718 | 3.34E-27 | 6.34E-26 |
| CASP8 | 1.376885 | 2.831424 | 1.040119 | 1.52E-15 | 4.81E-15 |
| TRAF2 | 2.172886 | 7.704197 | 1.826032 | 3.25E-27 | 6.34E-26 |
| USP22 | 6.075269 | 15.05483 | 1.309206 | 2.18E-19 | 1.24E-18 |
| SQSTM1 | 29.98683 | 99.91757 | 1.736409 | 9.02E-17 | 3.21E-16 |
| DNMT1 | 1.168587 | 4.380206 | 1.906233 | 7.50E-21 | 4.75E-20 |
| ID1 | 45.58779 | 19.75054 | -1.20676 | 1.65E-14 | 4.08E-14 |
| CDKN2A | 0.162719 | 4.09759 | 4.65432 | 1.87E-25 | 1.52E-24 |
| HSPA4 | 10.40937 | 24.42102 | 1.230241 | 4.84E-26 | 6.25E-25 |
| PLK1 | 0.135939 | 2.758213 | 4.342699 | 1.55E-27 | 6.34E-26 |
| MYCN | 0.053057 | 1.101482 | 4.375771 | 4.27E-15 | 1.22E-14 |
| TERT | 0.001912 | 1.369339 | 9.483907 | 5.48E-26 | 6.25E-25 |
| SLC39A7 | 25.43712 | 70.23569 | 1.465269 | 1.07E-25 | 1.02E-24 |
| RNF31 | 1.060268 | 2.220226 | 1.066278 | 2.34E-13 | 5.33E-13 |
| HSP90AA1 | 68.1895 | 141.9497 | 1.057758 | 6.55E-19 | 3.36E-18 |
| LEF1 | 0.118922 | 1.409514 | 3.567111 | 3.47E-17 | 1.32E-16 |
| TNFRSF21 | 3.951794 | 9.953828 | 1.332744 | 0.000119 | 0.000174 |
